# Chemical Immobilization of Carboxymethyl Chitosan on Polycaprolactone Nanofibers as Osteochondral Scaffolds

**DOI:** 10.1007/s12010-022-03916-6

**Published:** 2022-04-30

**Authors:** Anita Kabirkoohian, Hadi Bakhshi, Shiva Irani, Fereshteh Sharifi

**Affiliations:** 1 Department of Biology, Science and Research Branch, Islamic Azad University, Tehran, Iran; 2 Department of Life Science and Bioprocesses, Fraunhofer Institute for Applied Polymer Research IAP, Geiselbergstraße 68, 14476 Potsdam-Golm, Germany; 3 Hard Tissue Engineering Research Center, Tissue Engineering and Regenerative Medicine Institute, Central Tehran Branch, Islamic Azad University, Tehran, Iran

**Keywords:** Polycaprolactone, Carboxymethyl chitosan, Nanofibers, Immobilization, Osteochondral tissue engineering

## Abstract

Carboxymethyl chitosan (CMC) as a bio-based osteochondral inductive material was chemically immobilized on the surface of polycaprolactone (PCL) nanofibers to fabricate scaffolds for osteochondral tissue engineering applications. The chemical immobilization process included the aminolysis of ester bonds and bonding of the primary amines with glutaraldehyde as a coupling agent. The SEM and FTIR results confirmed the successfulness of the CMC immobilization. The fabricated scaffolds presented cell viabilities of > 82% and supported the attachment and proliferation of the human bone marrow mesenchymal stem cells (hBM-MSCs). The CMC-immobilized scaffolds concentration dependently induced the diverse osteochondral differentiation pathways for the hBM-MSCs without using any external differential agents. According to the Alcian Blue and Alizarin Red staining and immunocytochemistry results, scaffolds with a higher content of CMC presented more chondro-inductivity and less osteoinductivity. Thus, the CMC-immobilized scaffolds can be employed as great potential candidates for osteochondral tissue engineering applications.

**Supplementary information:**

The online version contains supplementary material available at 10.1007/s12010-022-03916-6.

## Introduction

The osteochondral defect is referred to as damage to the articular cartilage and the underlying subchondral bone, which results in mechanical instability of the joint or even osteoarthritis [1]. In the last years, the treatments for osteoarthritis are shifting from orthopedic surgery using synthetic implants or tissue grafting to osteochondral tissue engineering by employing biodegradable scaffolds loaded with biological molecules or cells to regenerate the tissues [2, 3]. Recently, there are many investigations on the development of osteochondral scaffolds to induce the simultaneous regeneration of articular cartilage and the subchondral bone [2–8]. Osteochondral scaffolds need unique structural and compositional properties with specific biological and mechanical to promote individual growth of both cartilage and bone cell layers within a single integrated implant.

The structure and composition of scaffolds are two determining factors during designing implants. Ideally, the scaffolds based on natural extracellular matrix (ECM) [9, 10] or components mimicking the structure and bio-functionality of the native ECM [11, 12], to physically guide or chemically inform cell response and thus promote tissue growth, are ideal for tissue engineering application. Carboxymethyl chitosan (CMC), obtained from the carboxymethylation of chitosan, is a biopolymer known to be biocompatible, biodegradable, water-soluble, and calcium-chelatable. Meanwhile, the molecular structure of CMC is similar to glycosaminoglycans (GAGs), linear heteropolysaccharides based on nonsulfated or sulfated disaccharide units [13]. GAGs such as chondroitin sulfate, keratan sulfate, and hyaluronan are articular cartilage compounds with important physiological functions [14, 15]. Therefore, osteochondral scaffolds based on CMC can expose more bioactivity and consequently enhance the tissue regeneration process [16].

Recently, we focused on employing CMC for the fabrication of electrospun scaffolds for bone [13, 17–20] and cartilage [21] tissue engineering applications, either through electrospinning of blend solutions or surface treatments as post-processes. Polycaprolactone (PCL) is one of the most common polymers for medical purposes due to its biocompatibility, biodegradability, toughness, easy processability, etc. PCL is frequently employed for bone and cartilage tissue engineering applications [8, 22]. The major disadvantage of PCL is the lack of surface hydrophilicity/wettability, which influences the cell attachment and consequently their proliferation and differentiation, essential in tissue engineering [23–25]. Here, we immobilized CMC on the surface of PCL nanofibers through the chemical grafting reaction to simply fabricate novel scaffolds, which can induce osteochondral differentiation on stem cells without using any external differential agents. The grafting was done through aminolysis of the ester bonds within the PCL backbone and then coupling with a dialdehyde. The morphology and biocompatibility of the fabricated scaffolds were evaluated. The activity of the immobilized CMC on the surface PCL nanofibers as a scaffold for osteochondral tissue engineering applications was studied. Human bone marrow mesenchymal stem cells (hBM-MSCs) were cultured on the scaffolds for the in vitro osteochondral differentiation study over 21 days.

## Experimental

All experimental details including the fabrication of scaffolds, instruments, and biological assays are provided in the supplementary information.

## Results and Discussion

### Fabricating PCL Scaffolds

**Fig. 1:**
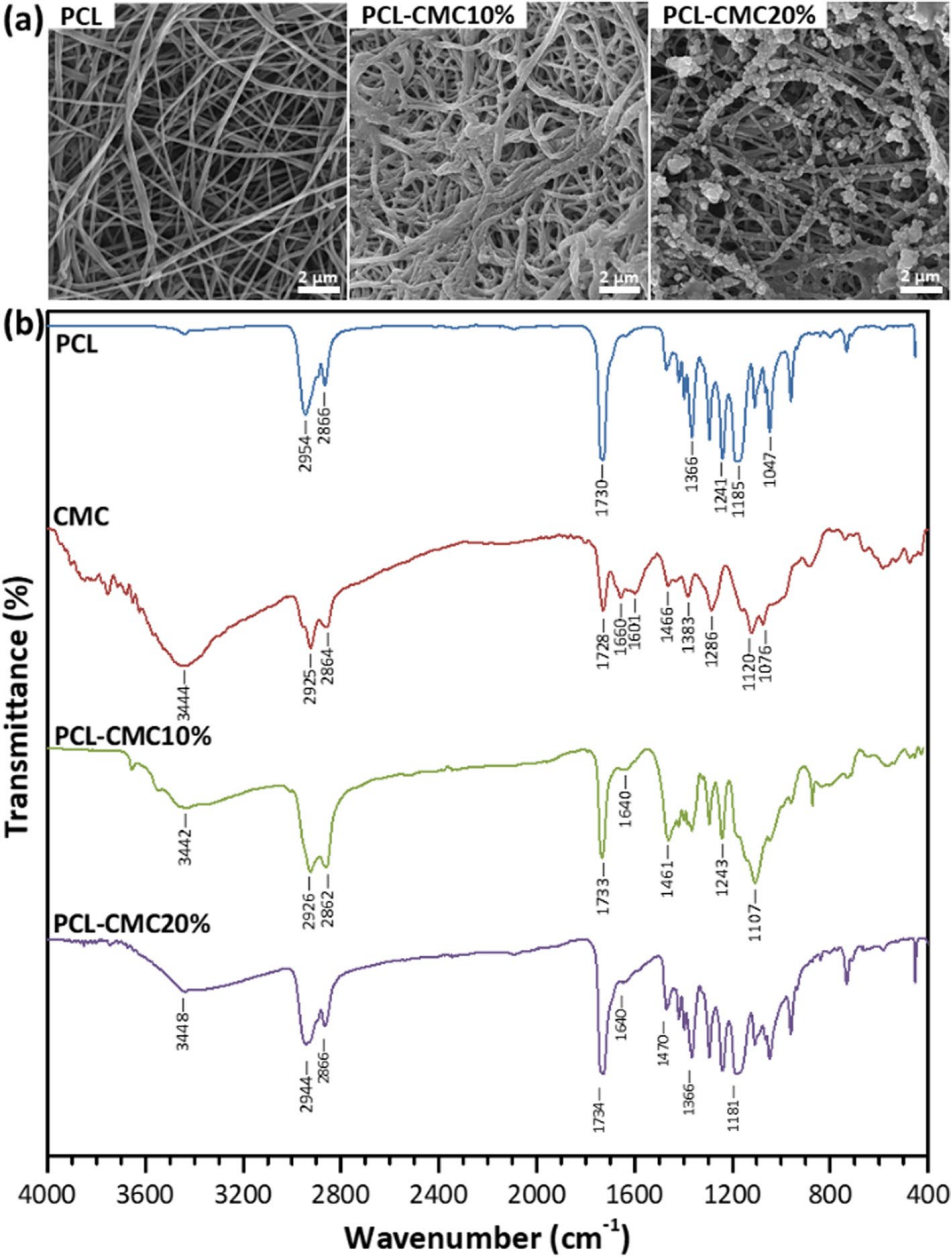
(**a**) SEM images of the scaffolds. (**b**) FTIR spectra for the PCL scaffold, CMC, and the CMC-immobilized scaffold

In the last decades, several techniques such as freeze-drying, electrospinning, salt-leaching, and nanotopography modulating are being utilized to fabricate scaffolds with high surface areas for tissue engineering applications [26]. In the meantime, the electrospinning process is a powerful technique to fabricate nanofibrous structures with high porosity, similar to the extracellular matrix (ECM), from synthetic and natural biomaterials [27, 28]. Here, the PCL nanofibers were fabricated through the electrospinning of PCL solution (*M*_*n*_ = 80,000 g/mol, 12.5 wt%) in a mixture of acetic acid/formic acid (2/3, v/v). The concentration of PCL solution of 12.5 wt% was obtained through the previous optimizations regarding the electrospinnability and narrower diameter of nanofibers. SEM images showed uniform, random-oriented, and bead-less PCL nanofibers with an average diameter of 162 ± 39 nm and smooth surfaces (Fig. 1a). The electrospun PCL mats presented large interconnected cavities, which are well-suited to provide the appropriate biological conditions for seeded cells.

**Scheme 1:**
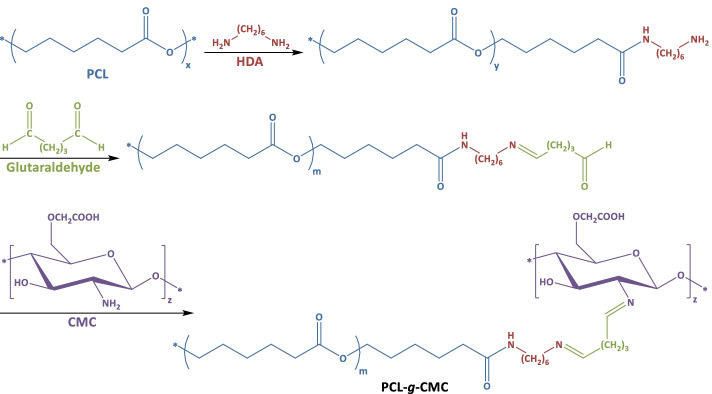
Reaction route for the chemical immobilization of CMC on the surface of PCL nanofibers

Different strategies have been considered to incorporate CMC within the electrospun scaffolds, namely blending electrospinning [20] or chemical immobilization on the surface of electrospun mats [17]. The immobilization strategy through chemical bonding not only has no side effects on the mechanical properties of the scaffold bulk material but also provides the possibility for co-immobilization of other bioclues and proteins. Here, the surface of PCL nanofibers was chemically immobilized with CMC. For this purpose, the ester bonds of PCL chains were aminolized with 1,6-hexamethylenediamine (HAD) to introduce free primary amine groups on their surfaces (Scheme 1). Later, these primary amine functions were bonded to the amine groups of CMC using glutaraldehyde as the coupling agent [17]. Through employing two CMC solutions with different concentrations (10% or 20%), scaffolds with two levels of immobilized CMC on their surfaces (PCL-CMC10% and PCL-CMC20%, respectively) were prepared. The concentration of CMC solution of 20% was the highest processable concentration regarding the viscosity.

The immobilization of CMC on the surface of PCL nanofibers was studied by FTIR spectroscopy (Fig. 1b). The FTIR spectrum of the PCL-CMC10% and PCL-CMC20% scaffolds displayed the characteristic peaks of CMC (1601, 1660, and 3444 cm^− 1^) arising from the stretching vibration of C = O, O‒H, and N‒H bonds) [17, 20, 21] comparing to the spectrum for the PCL scaffold (1045, 1185, and 1730 cm^− 1^) [29], which confirmed the successful chemical immobilization process. The morphology of the CMC-immobilized scaffolds was investigated by SEM (Fig. 1a). Results showed that CMC was more uniformly immobilized on the surface of nanofibers within the PCL-CMC10% scaffold compared to the PCL-CMC20% one, where CMC particles with a diameter of 200–500 nm were generated in course of the immobilization process. The average diameters of nanofibers for the PCL-CMC10% and PCL-CMC20% scaffolds were 240 ± 80 and 250 ± 142 nm, respectively, which showed thicknesses of 78 and 88 nm, respectively, for the immobilized CMC layers.

### Biocompatibility

**Fig. 2:**
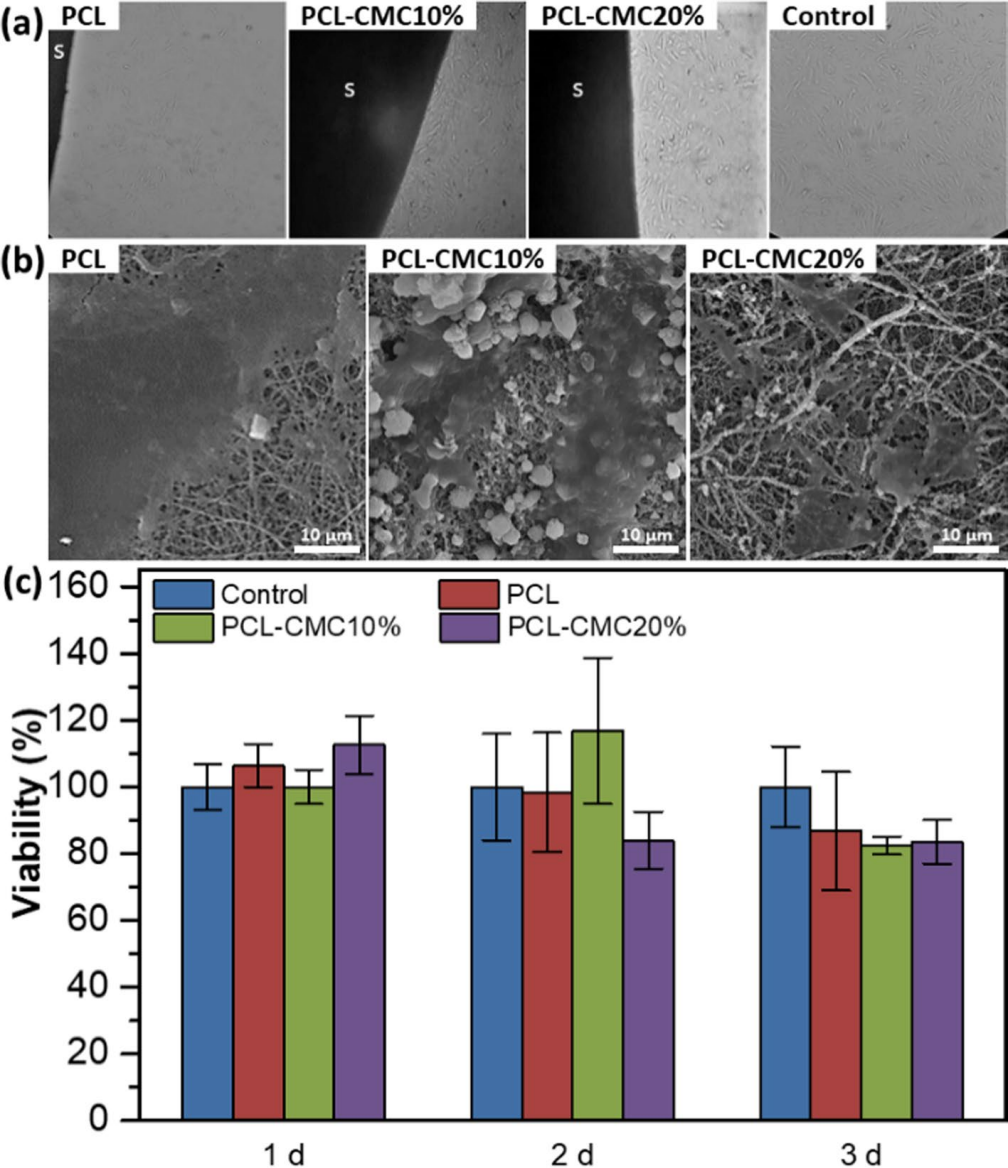
Optical microscopic (**a**) and SEM (**b**) images of the hBM-MSCs (10^4^ cells) seeded on the scaffolds (0.5 × 0.5 cm^2^) after 3 days of incubation. A tissue culture plate was used as the control. The magnification is 100. S shows the scaffolds. (**c**) The viability of the hBM-MSCs on the scaffolds up to 3 days of incubation obtained by MTT assay (*n* = 3)

Biocompatibility is the critical trait of tissue engineering scaffolds for cellular attachment, proliferation, and differentiation. Thus, the biocompatibility of the fabricated scaffolds was determined by the direct contact method. For this purpose, hBM-MSCs (10^4^ cells, passaged three times) were seeded on the scaffolds (0.5 × 0.5 cm^2^), and their viability was evaluated up to 3 days of incubation. The optical microscopy images displayed that the spindle-shaped hBM-MSCs have proliferated and moved toward the scaffolds after 3 days of incubation (Fig. 2a). The SEM images showed a good spread and attachment of the seeded hBM-MSCs on the scaffolds, where well-developed cell-cell and cell-matrix interactions with actin filaments (invadopodia and filopodia) were observed (Fig. 2b).

According to the MTT results, no cytotoxicity effects were detected for the scaffolds for up to 3 days, where cell viabilities of > 82% were observed (Fig. 2c). Meanwhile, the cell viabilities of the hBM-MSCs on the scaffolds were not significantly different (*p *> 0.5) compared to the tissue culture plate used as the control. These results confirmed that the fabricated scaffolds are biocompatible and can support the attachment, proliferation, and differentiation of the stem cells.

### Osteochondral Differentiation

MSCs, as the main source of adult stem cells, have found several applications in cell therapy and tissue engineering as a result of their ability to self-reproduce and differentiate into a range of cells and later tissues such as bone, cartilage, muscle, tendon, ligament, and fat [27]. Stem cells seeded in a scaffold can differentiate into chondroblasts/chondrocytes or osteoblasts/osteocytes either directly or indirectly with the help of growth factors such as hormones and cytokines. The growth factors trigger the cartilage/bone creation by placement on their special receptors. Tissue engineering scaffolds based on CMC play an inducer role in chondrogenic [21] and osteogenic [13, 17, 19, 20] differentiation without using any external differential agents. It is due to the structural similarity of CMC to various GAGs present in the osteochondral ECM [13]. Meanwhile, the carboxyl acid groups within CMC can chelate with calcium ions. Hence, a CMC-riched scaffold can induce calcium phosphate formation, which considerably enhances its bioactivity [17].

**Fig. 3:**
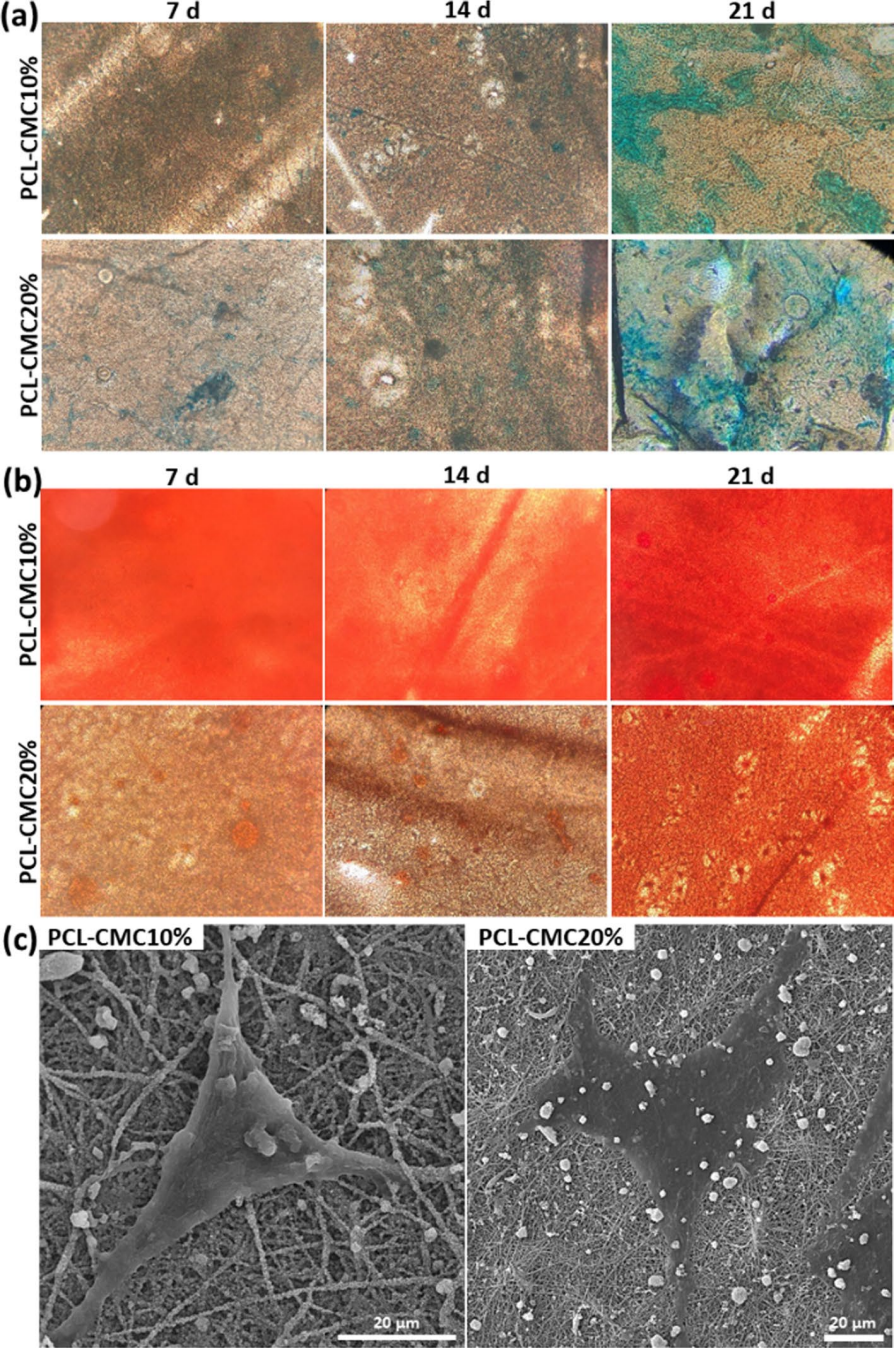
Alcian Blue (**a**) and Alizarin Red (**b**) staining of the scaffolds (0.5 × 0.5 cm^2^) seeded with hBM-MSCs (10^4^ cells) for up to 21 days of incubation. The magnification is 200. (**c**) SEM images of cells on the scaffolds after 7 days of incubation

To study the osteochondral inductivity of the immobilized CMC, hBM-MSCs (10^4^ cells, passaged three times) were seeded on the PCL-CMC10% and PCL-CMC20% scaffolds (0.5 × 0.5 cm^2^) and cultured in the nutrient medium without an external differential agent up to 21 days. Alcian Blue staining was performed to qualitatively evaluate the chondro-differentiation of the seeded hBM-MSCs on the scaffolds (Fig. 3a). Alcian Blue dye selectively attaches to the proteoglycan regions of the external chondrocyte matrix and changes the color of the chondrocytes to the blue [21]. The pH of the Alcian Blue solution was adjusted to 2.5 to just stain the GAGs generated through the chondrogenesis process [21, 30]. The progressive intensification of the blue color on the scaffolds indicated the gradual maturation of the seeded hBM-MSCs to chondrocytes. This phenomenon was in agreement with previous observations about the chondroinductivity of CMC [21]. The PCL-CMC20% scaffold demonstrated more absorption of Alcian Blue dye compared to the PCL-CMC10% one, especially after 7 and 14 days of incubation, which means its better chondro-differentiation inductivity.

The osteo-differentiation of the seeded hBM-MSCs on the scaffolds was qualitatively assessed by Alizarin Red staining (Fig. 3b). Alizarin Red dye is selectively absorbed by the mineralized matrix and changes the color of the scaffolds to red [27, 28]. The results exhibited the absorption of Alizarin Red dye on the scaffolds, particularly on PCL-CMC10% even after 7 days of incubation, due to the calcium deposed by osteo-differentiated cells. This observation was in agreement with previous reports about the osteoinductivity of CMC [13, 17, 19, 31]. CMC as a bioactive component similar to GAGs in the hard tissue, in particular bone, induces the formation of CaPO_4_ or hydroxyapatite and consequently promotes osteogenesis [20, 32]. The intensity of absorbed dye on the PCL-CMC10% scaffold was higher than the PCL-CMC20% one, which indicated its superior osteo-differentiation inductivity. However, the absorption of Alizarin Red dye on the PCL-CMC20% scaffold was increased over 21 days, indicating the progress of osteo-differentiation of the stem cells during this period.

The morphology of the seeded cells was monitored through SEM (Fig. 3c). The hBM-MSCs cultured on the PCL-CMC10% scaffold for 7 days displayed a shape similar to osteoblasts, while the cells on the PCL-CMC20% scaffold exhibited the morphology of chondrocytes. Meanwhile, the SEM images showed that the seeded cells uniformly distributed and penetrated within the scaffolds, which confirmed the excellency of the CMC-immobilized scaffolds for cell adhesion, proliferation, and differentiation.

**Fig. 4:**
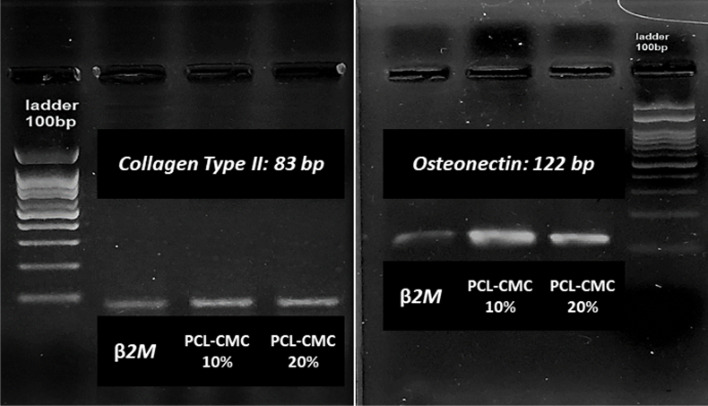
RT-PCR results for expression of Collagen Type II and Osteonectin genes for osteochondral differentiated cells (10^6^ cells) on scaffolds (0.5 × 0.5 cm^2^) after 21 days of incubation. The expression of β2M was recorded as a control gene

The osteochondral inductivity of the CMC-immobilized scaffolds was evaluated by assessing the expression of *Collagen Type II* and *Osteonectin* genes in mRNA levels for the seeded cells through reverse transcription-polymerase chain reaction (RT-PCR). For this purpose, hBM-MSCs (10^6^ cells, passaged three times) were seeded on the PCL-CMC10% and PCL-CMC20% scaffolds (0.5 × 0.5 cm^2^) and kept in the nutrient medium without an external differential agent for 21 days. Then, the total RNA of the cells was isolated and converted into cDNA for the RT-PCR process (Fig. 4). The expression of β2M was recorded as a control gene. About 50–70% of the cartilage cell matrix is composed of collagen; hence, *Collagen Type II* (band at 83 bp), known as the most important gene for following the chondrogenesis process of stem cells [21], was detected for the cells culture on both PCL-CMC10% and PCL-CMC20% scaffolds after 21 days of incubation. This phenomenon was in agreement with previous observations about the chondroinductivity of CMC [21]. *Osteonectin* (band at 122 bp), a marker gene for the osteogenesis process [19, 27, 28], was also expressed for both PCL-CMC10% and PCL-CMC20% scaffolds after 21 days of incubation. This observation was in agreement with previous reports regarding the osteoinductivity of CMC [13, 17, 19, 31]. It means that the immobilized CMC on the PCL scaffolds successfully induced osteochondral differentiation for the seeded hBM-MSCs without using any external differential agents.

**Fig. 5:**
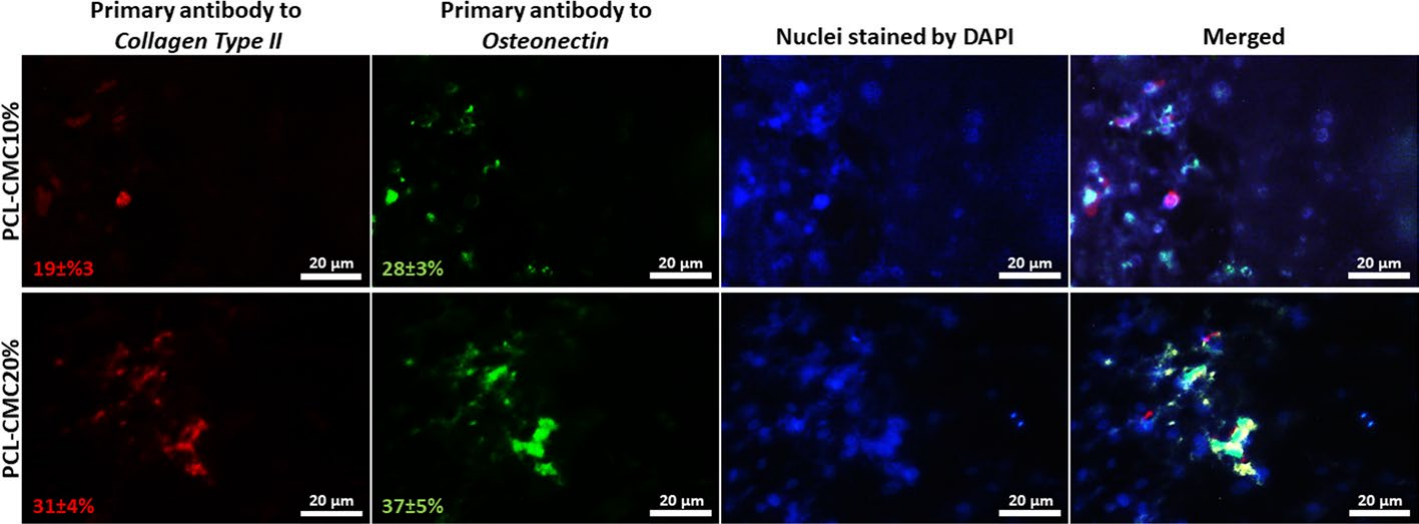
ICC results for expression of Collagen Type II and Osteonectin proteins for osteochondral differentiated cells (10^4^ cells) on scaffolds (0.5 × 0.5 cm^2^) after 21 days of incubation (*n* = 3)

Finally, the expression of *Collagen Type II* and *Osteonectin* proteins for the seeded cells was investigated by immunocytochemistry (ICC) assay. To this end, hBM-MSCs (10^4^ cells) were seeded on the PCL-CMC10% and PCL-CMC20% scaffolds (0.5 × 0.5 cm^2^) and cultured in the nutrient medium without an external differential agent for 21 days. The fluorescence microscopy images exhibited the expression of both *Collagen Type II* and *Osteonectin* proteins for both PCL-CMC10% and PCL-CMC20% scaffolds (Fig. 5). According to the fluorescence microscopy images (*n *= 3), the ratio of *Collagen Type II* to *Osteonectin* expressions for the cells on the PCL-CMC10% scaffold was 19% / 28% = 0.68, while this ratio for the cells on the PCL-CMC20% scaffold was 31% / 37% = 0.84. It means that the PCL-CMC20% scaffold was more chondro-inductive and less osteo-inductive than the PCL-CMC10% one, which was in agreement with the Alcian Blue and Alizarin Red staining results (Fig. 3a-b). The hBM-MSCs seeded on the scaffolds successfully underwent osteochondral differentiation by the immobilized CMC as an inducer without using any external differential agents.

## Conclusions

CMC can be chemically immobilized on the surface of PCL nanofibers through aminolysis and glutaraldehyde-coupling processes, to simply fabricate scaffolds for osteochondral tissue engineering applications. The SEM and FTIR results confirmed the successfulness of the chemical immobilization process. The MTT results showed that the fabricated scaffolds are biocompatible and support the attachment and proliferation of the seeded hBM-MSCs (cell viability of > 82%). The CMC-immobilized scaffolds could provide a suitable microenvironment for inducing the diverse osteochondral differentiation pathways for the hBM-MSCs without using any external differential agents. The osteochondral inductivity of the CMC-immobilized scaffold was concentration-dependent. According to the Alcian Blue and Alizarin Red staining and ICC results, increasing the content of immobilized-CMC (PCL-CMC20%) resulted in more chondro-inductivity and less osteoinductivity. As a research limitation, observing the complete differentiation of hBM-MSCs into chondrocytes and osteocytes was not possible in this in vitro study; although the osteochondral differentiation of the stem cells seeded on the CMC-immobilized scaffolds for 21 days was confirmed, however, an in vivo study could be very beneficial. Meanwhile, the osteochondral differentiation could be more quantitatively evaluated through the calcium content and the real-time PCR assays. Moreover, this study can be extended by exploring the mechanisms of the osteochondral differentiation of the hBM-MSCs induced by immobilized CMC moieties.

## Supplementary Information

Below is the link to the electronic supplementary material.

ESM 1 (PDF 879 KB)

## Data Availability

All data generated or analyzed during this study are included in the manuscript and the supplementary information.
