# Supplementary material for: Chemical Immobilization of Carboxymethyl Chitosan on Polycaprolactone Nanofibers as Osteochondral Scaffolds
Source: Appl Biochem Biotechnol. 2022 Apr 30;195(6):3888–99. doi: 10.1007/s12010-022-03916-6 (PMC10203026; doi:10.1007/s12010-022-03916-6)
Supplement: Supplementary file 1 — (PDF 879 KB) [file 12010_2022_3916_MOESM1_ESM.pdf]

Supplementary Information

For

Chemical Immobilization of Carboxymethyl Chitosan on Polycaprolactone Nanofibers as Osteochondral Scaffolds

Anita Kabirkoohian[a], Hadi Bakhshi*[,b], Shiva Irani[a], Fereshteh Sharifi[c]

[a]Department of Biology, Science and Research Branch, Islamic Azad University, Tehran, Iran.
[b]Department of Life Science and Bioprocesses, Fraunhofer Institute for Applied Polymer Research IAP, Geiselbergstraße 68, 14476, Potsdam-Golm, Germany.
[c]Hard Tissue Engineering Research Center, Tissue Engineering and Regenerative Medicine Institute, Central Tehran Branch, Islamic Azad University, Tehran, Iran.
*Corresponding author: Hadi Bakhshi, E-mail address: hadi.bakhshi@iap.fraunhofer.de

1 Experimental

1.1 *Materials*

Chitosan extracted from the crab shell with a medium weight and degree of deacetylation of >90% was purchased from Bio Basic (Canada). Polycaprolactone (PCL) with an average molecular weight (M_n) of 80,000, 1,6-hexamethylenediamine (HDA), 3-[4,5-dimethylthiazol-2-yl]-2,5-diphenyltetrazolium bromide (MTT), 4',6-diamidino-2-phenylindole (DAPI), Alcian Blue, Alizarin Red, Syber Green, *Osteonectin* antibody, and dimethyl sulfoxide (DMSO) were obtained from Sigma-Aldrich (Germany). Hydrochloric acid, formic acid, acetic acid, monochloroacetic acid, sodium hydroxide, glutaraldehyde, ethanol, and isopropanol were supplied by Merck (Germany). Dulbecco's modified eagle's medium (DMEM), fetal bovine serum (FBS), phosphate-buffered saline (PBS), and trypsin/EDTA solution (0.25%) were bought from Gibco (USA). *Collagen II* antibody and secondary antibody were purchased from Abcam (USA).

1.2 *Synthesis of carboxymethyl chitosan (CMC)*

Carboxymethyl chitosan was synthesized according to our previous report [1]. Briefly, Chitosan (1 g) was purified by dissolving in acetic acid solution (1%, 40 mL) at room temperature, precipitating with sodium hydroxide solution (1 M, 50 mL), and washing with deionized water and later isopropanol. The purified chitosan was added to sodium hydroxide solution in isopropanol (0.1 g/mL, 20 mL) and mechanically stirred for 5 h to completely be dissolved. Afterward, a monochloroacetic acid solution in isopropanol (0.4 g/mL, 5 mL) was added dropwise to the chitosan solution and stirred at room temperature for 8 h. Finally, the resulting precipitate was filtered, washed with a mixture of ethanol/deionized water (1/3, v/v) three times, and dried at room temperature in a vacuum oven.

1.3 *Electrospinning PCL nanofibers*

The PCL nanofibers were fabricated through the electrospinning process. PCL solution (12.5 wt%) in a mixture of acetic acid/formic acid (2/3, v/v) was electrospun on an Asia Nanostructure apparatus (CO881007NYI, Iran) operating at 30 °C with a flow rate range of 0.1 mL/h and a voltage of +25 kV. The PCL fibers were collected on aluminum sheets rating at 250 rpm at a distance of 15 cm to the needle for 60 min. The electrospun mats were cut in 0.5×0.5 cm^2 dimensions before use.

1.4 *Chemical immobilization of CMC on PCL nanofibers*

The PCL nanofibers (0.5×0.5 cm^2) were initially immersed in a mixture of ethanol/deionized water (1/1, v/v, 500 µL) and then washed with deionized water to eliminate any contamination. The chemical grafting of CMC on the surface of the PCL nanofibers was done in four steps. In the first step, The PCL nanofibers were immersed in HDA solution (50 g/L, 100 µL) in isopropanol/deionized water (9/1, v/v) at room temperature for 4 h and washed with deionized water. In the second step, the PCL nanofibers were immersed in glutaraldehyde solution in PBS (1%, 500 µL) at room temperature for 4 h and washed with deionized water. In the third step, the PCL nanofibers were immersed in CMC solution in PBS

(10% or 20%, respectively 100 g/L or 200 g/L, 50 µL) at room temperature for 24 h, washed with deionized water, and dried in a vacuumed oven overnight. The scaffolds were named PCL-CMC10% and PCL-CMC20%, respectively.

1.5 Characterization of scaffolds

To evaluate the chemical grafting progress, Fourier-transform infrared spectroscopy (FTIR) spectroscopy was done on a Perkin Elmer (Spectrum RX I, USA) in a range of 400-4000 cm^{-1} with a resolution of 4 cm^{-1}. The morphology of scaffolds was studied through scanning electron microscopy (SEM, Tescan, Vega II, Czech Republic). All specimens were coated with a gold layer before imaging. The diameter of fibers was measured using Image J software (version 1.41).

1.6 Cell Seeding of scaffolds

Human bone marrow mesenchymal stem cells (hBM-MSCs) obtained from the Stem Cell Technology Research Center (Tehran, Iran) were cultured in DMEM medium supplemented with FBS (10%), penicillin (100 µg/mL), and streptomycin (100 µg/mL) in a humidified incubator at 37 °C under CO_2 (5%). The cells were passaged three times before seeding on the scaffolds. Each side of the scaffolds was sterilized under UV radiation for 20 min. The cell suspension (100 µL, 10^4 or 10^6 cells) was placed on the scaffold (0.5×0.5 cm^2) in a 96-well tissue culture plate in triplicate and incubated at 37 °C under CO_2 (5%) for 2 h for the attachment of cells. Finally, the seeded scaffolds were cultured in DMEM (1 mL) supplemented with FBS (10%) in an incubator at 37 °C under CO_2 (5%). The media were replaced every three days. A tissue culture plate without any scaffold was used as a control.

1.7 Biocompatibility assays

The morphological changes of the seeded cells (10^4 cells/0.5×0.5 cm^2 scaffold) up to 7 d of incubation were studied under an optical microscope (Olympus, Japan) after 24-72 h of incubation.

The proliferation of cells (10^4 cells/0.5×0.5 cm^2 scaffold) up to 21 d of incubation was determined through MTT assay. For this purpose, the medium of each sample (n=3) was replaced with 200 µL of MTT solution (5 g/L) followed by incubation at 37 °C for 3 h. The formed formazan crystals were dissolved in DMSO and the optical density (OD) of the solution was measured at 570 nm on an Eliza reader instrument (Bio-Tek ELx 800).

The morphology and attachment of the seeded cells (10^4 cells/0.5×0.5 cm^2 scaffold) up to 14 d of incubation were studied by SEM. To this end, the cells were washed with PBS and fixed in a glutaraldehyde solution (2.5%), dehydrated in a series of ethanol solutions (60%, 70%, 80%, 90%, and 100%), and dried at room temperature.

The healthiness of seeded cells (10^4 cells/0.5×0.5 cm^2 scaffold) after 21 d of incubation was evaluated through DAPI staining. For this purpose, the cells were washed with PBS, fixed in a paraformaldehyde solution (4%), immersed in Triton X-100 solution (0.3%), and washed

again with PBS. Finally, the nucleus of the fixed cells was stained with DAPI solution (20 g/L) in darkness and observed under a confocal fluorescent microscope (Labomed, LX400, USA).

1.8 Differentiation assays

The chondro-differentiated cells contain glycosaminoglycans (GAGs). Therefore, Alcian Blue staining was used to visualize the GAGs content of cells up to 21 d of incubation. For this purpose, the seeded cells (10^4 cells/0.5×0.5 cm^2 scaffold) were washed with PBS, fixed with glutaraldehyde solution (4%), and again washed with PBS. The fixed cells were stained with Alcian Blue solution (10 g/L, 100 µL) in acetic acid (3%) for 45 min, destained in HCL solution (0.1 N) for 3 min, and washed twice with PBS. Finally, the stained cells were observed under an inverted microscope (Olympus, Japan).

The osteo-differentiated cells generate calcium on the scaffolds. Therefore Alizarin Red staining was employed to study the calcium deposits by cells up to 21 d of incubation. To this end, the seeded cells (10^4 cells/0.5×0.5 cm^2 scaffold) were washed with PBS, fixed with paraformaldehyde solution (10%), and again washed with distilled water. Finally, the fixed samples were stained with Alizarin Red solution (20 g/L, 100 µL) and observed under a reverse microscope (Olympus, Japan).

The expressions of chondrogenic gen (*Collagen Type II*) and osteogenic gen (*Osteonectin*) at the mRNA level in the seeded cells (10^6 cells/0.5×0.5 cm^2 scaffold) after 21 d of incubation were evaluated by reverse transcription-polymerase chain reaction (RT-PCR). After washing twice with PBS, the total RNA of the seeded cells was extracted by a total RNA isolation kit (Dena Zist Asia, Iran) and converted to cDNA using an easy cDNA synthesis kit (Parstous Biotechnology, Iran) according to the manufacturer's protocols. Finally, the RT-PCR process was performed on a thermal cycler (Rotor Gene 6000, Qiagen, USA) using a master mix reagent (2X PCR Master Mix, Biofact Co., South Korea). The primers designed for RT-PCR are shown in Table 1. The expression of *Beta-2 macroglobulin* (*β2M*) was evaluated as a reference gene.

Table 1. Sequences of the primers for the target genes

Gene	Primer
Collagen Type II	F: 5' GTC ACA GAG GTT ATC CAG 3'
	R: 5' ACC CGG GGA ACC ACT CTC 3'
Osteonectin	F: 5' ACA TCG GGC CTT GCA AAT AC 3'
	R: 5' GTT GTC CTC ATC CCT CTC AT 3'
β2M	F: 5' CCA CTG AAA AAG ATG AGT ATG CCT 3'
	R: 5' CCA ATC CAA ATG CGG CAT CTT CA 3'

The expressions of *Collagen Type II* and *Osteonectin* markers in the seeded cells (10^4 cells/0.5×0.5 cm^2 scaffold) after 21 d of incubation were assessed by immunocytochemistry

(ICC) assay. The seeded scaffolds were washed with PBS, fixed with paraformaldehyde solution (4%) at 4 °C for 20 min, washed with PBS, permeabilized with Triton X-100 (4%) for 10 min, and washed again with PBS. The permeabilized cells were immersed in FBS for 45 min to block the nonspecific binding sites, incubated with *Collagen Type II* or *Osteonectin* primary antibody (500 mg/L or 200 mg/L, respectively, Sigma-Aldrich, Germany) at 4 °C for 24 h, washed with PBS, incubated with secondary antibodies conjugated with phycoerythrin (Chemicon Temecula) at 37°C for 1 h, and washed again with PBS. Eventually, the nuclei of cells were stained with DAPI and observed under a confocal fluorescent microscope (Labomed, PCM400, Denmark).

1.9 Statistical analysis

The results expressed as mean ± SD are representing at least three independent experiments. The differences between groups were analyzed using the one-way ANOVA method after testing for homogeneity of variances by the PASW Statistics program package (version 19, SPSS Inc., USA). The statistical significance was assigned as * for $p \leq 0.05$, ** for $p \leq 0.01$, and *** for $p \leq 0.001$.

References

1. Sharifi, F., Atyabi, S. M., Norouzian, D., Zandi, M., Irani, S., & Bakhshi, H. (2018). Polycaprolactone/carboxymethyl chitosan nanofibrous scaffolds for bone tissue engineering application. *International journal of biological macromolecules*, *115*, 243–248.
